# BRIP-1 germline mutation and its role in colon cancer: presentation of two case reports and review of literature

**DOI:** 10.1186/s12881-019-0812-0

**Published:** 2019-05-07

**Authors:** Mir Ali, Celia Dawn Delozier, Uzair Chaudhary

**Affiliations:** San Francisco-Fresno (UCSF Fresno), University of California, 155 N Fresno St, Fresno, CA 93701 USA

**Keywords:** Colorectal Cancer, Germline mutation, BRIP1 gene, Case report

## Abstract

**Background:**

Hereditary colon cancer is characterized by the inheritance of an abnormal gene mutation which predisposes to malignancy. Recent advances in genomic medicine have identified mutations in “novel” genes as conferring an increased risk of colorectal cancer. Mutations in the *BRIP1* gene (*BRCA1* Interacting Protein C- terminal helicase 1) are known to increase the risk of ovarian and breast cancers, but this genes association with colon cancer has not been previously reported.

**Case presentation:**

We describe two patients with colon cancer whose tumor tissue were found to harbor *BRIP1* mutations on analysis by next-generation sequencing. These patients were confirmed by analysis of lymphocytes to carry the mutation in the germline as well.

**Conclusions:**

These case reports highlight a previously unreported association of *BRIP1* germline mutations with colon cancer predisposition.

## Background

Colon cancer is the third most common cause of cancer-related mortality in the United States [1]. Between 2 to 5% of all colon cancers arise in the setting of well-defined inherited colon cancer syndromes including Lynch syndrome, Familial adenomatous polyposis, and *MUTYH*-associated polyposis. These syndromes are due to highly penetrant inherited gene mutations. An additional 20% of colorectal cancers are “familial” and likely linked to alterations in single genes that are less penetrant than those associated with well characterized syndromes [2]. Some of these gene mutations associated with an increased risk of digestive system cancers include *BMPR1A, CDH1, CHEK2, GREM1, POLD1, POLE, PTEN, SMAD4, TP53*. Patients and family members identified to have these germline mutations have an increased risk of colorectal/digestive system cancers. They benefit from early screening measures with colonoscopy and potential monitoring for other cancers associated with mutations in that specific gene. Similarly, the identification of additional new pathogenic mutations which may be involved in the development of colon cancer can provide us with valuable information for screening and patient counseling. We present two case reports and hypothesize that patients with *BRIP1 (BRCA1* Interacting Protein C- terminal helicase 1) gene mutations have an increased risk of colorectal cancer. *BRIP1* is a tumor suppressor gene interacting with another known DNA repair gene, *BRCA-1* (Breast Cancer gene 1), involved in repair by homologous recombination. Pathogenic germline mutations in *BRIP1* are known to confer about 10% cumulative risk of ovarian cancer and also associated with an increased risk of female breast cancer [3]. Based on our literature review, the association of *BRIP-1* mutations with colon cancer has not been previously reported. We describe here two patients with colon cancer who were found to have a *BRIP1* mutation in tumor tissue which was subsequently identified in the germline and postulate that germline *BRIP1* mutations confer an increased risk of colorectal cancer.

## Case presentation

### Case report 1

**Fig. 1:**
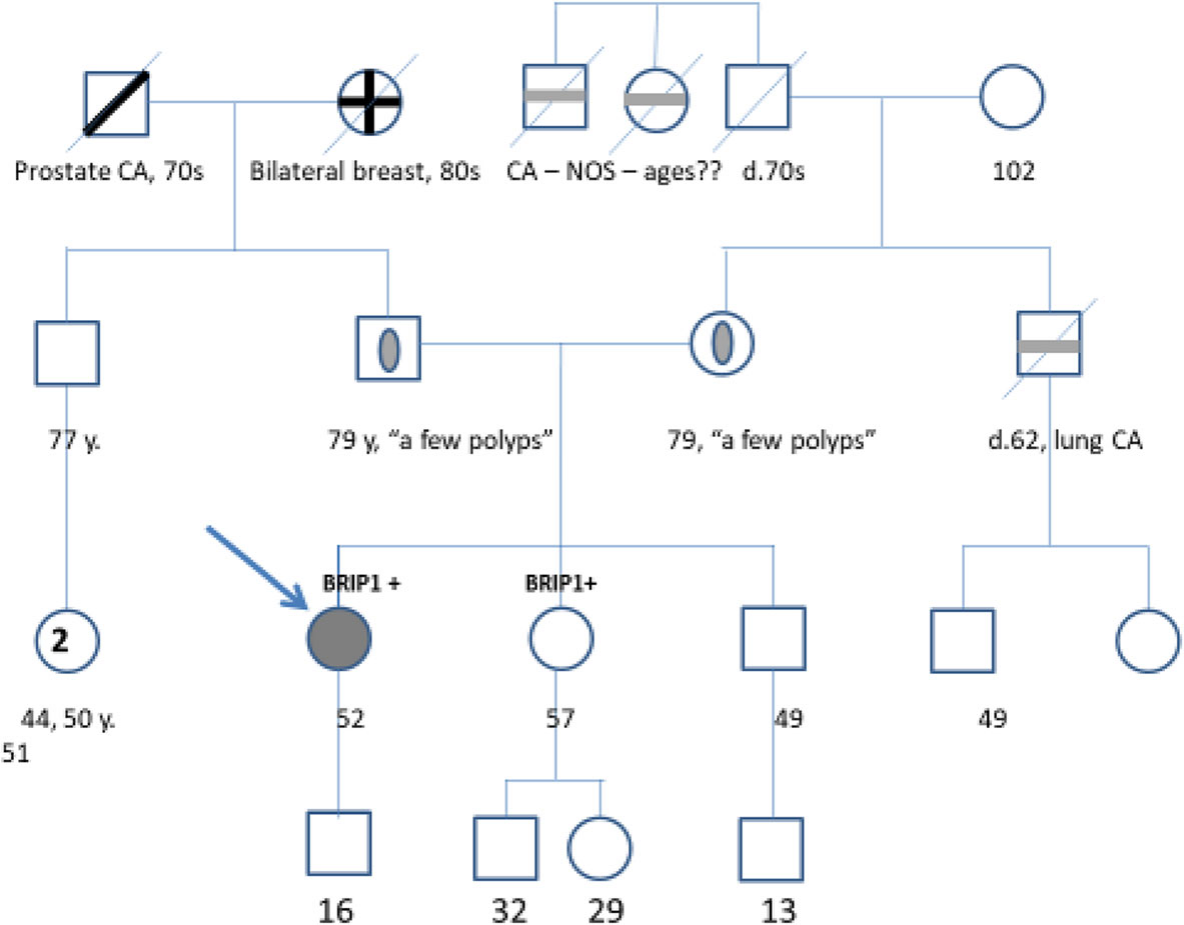
Family pedigree chart for case report 1

**Fig. 2:**
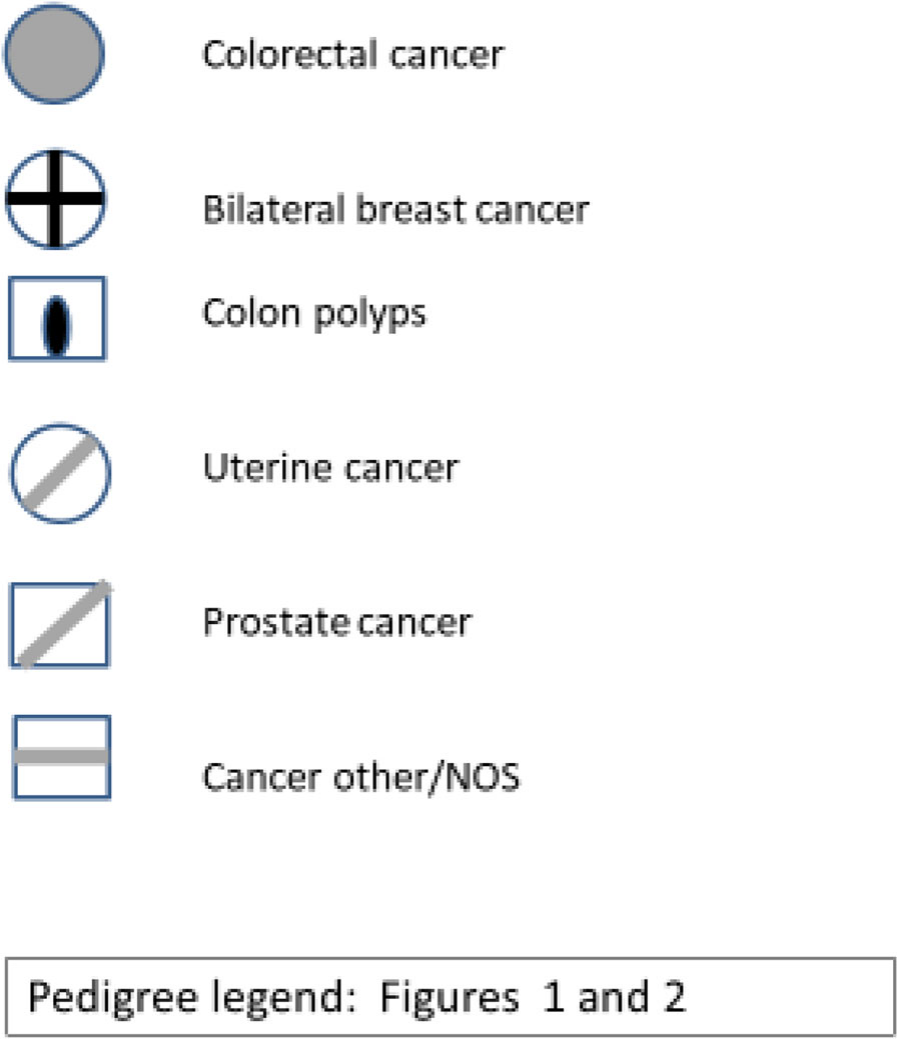
Pedigree legend for Figs. 1 and 3

52 y/o female with no significant past medical history initially presented with left lower quadrant abdominal pain. Family history includes lung cancer in brother at age 62 years who had a risk factor of chronic smoking. Paternal grandmother had bilateral synchronous breast cancer at the age of 80. Grandfather had prostate cancer at 79. No family history of gastrointestinal or ovarian cancer was reported. Pedigree chart for patient’s family history of cancer is shown below (Figs. 1 and 2). Physical examination was normal. CT scan of the abdomen showed an obstructed rectosigmoid mass. Colonoscopy showed recto sigmoid mass 4.5 × 6.5 cm which was 18 cm from the anal verge. Biopsy revealed a moderately differentiated adenocarcinoma. A CT scan of the chest and abdomen did not show evidence of distant metastases. The patient underwent laparoscopic rectosigmoid and local lymph node resection with a left end colostomy. Pathology showed an invasive, moderately-differentiated adenocarcinoma with infiltration beyond the muscularis propria into subserosal tissue. There were high risk features, including lymphovascular, perineural invasion along with six of twenty-eight lymph nodes positive for adenocarcinoma. Initial surgical specimen after colectomy was sent for analysis with next generation sequencing test. Tumor mutations included BRIP1 P619Fs*20: TP53 S2151, splice site *783-2A > G: CDK8* amplification was equivocal and APC E 1295*. Lynch syndrome screen by immunohistochemistry (*MLH1, MSH2, MSH6* and *PMS2* proteins) showed normal expression in pathological tissue. There were no reportable alterations in *KRAS, NRAS,* and *BRAF*. The patient had colon cancer at the relatively young age of 52 with no family history. The patient requested an evaluation of possible hereditary predisposition. Initial testing with a commercially available 17 gene colon cancer risk panel was negative. Additional germline testing for *BRIP1* gene mutation was proposed based on high allele frequency of *BRIP1* mutation in tumor tissue. The patient was found to be heterozygous for the *c.853_1854lnsG* mutation located in coding exon 12 of the *BRIP1* gene. No other germline mutations were identified with the panel testing used. She was staged as IIIB disease (pT3 pN2a M0). She was treated with twelve cycles of adjuvant chemotherapy FOLFOX (5-fluorouracil, leucovorin, oxaliplatin). Six months after finishing chemotherapy, the patient underwent laparotomy for planned colostomy reversal. She was found to have intraoperative findings of metastasis to the omentum and left the ovary. She was treated with HIPEC surgery (Hyperthermic intraperitoneal chemotherapy), omentectomy and bilateral salpingo-oophorectomy. She then received eight cycles of chemotherapy FOLFIRI (5-fluorouracil, Leucovorin, Irinotecan) for disease recurrence. The patient had another recurrence 1 year later with new pulmonary metastatic disease and pleural effusion. She was treated with six cycles of chemotherapy with FOLFOX and Bevacizumab and currently on maintenance 5-Fluorouracil and Bevacizumab therapy.

### Case report 2

**Fig. 3:**
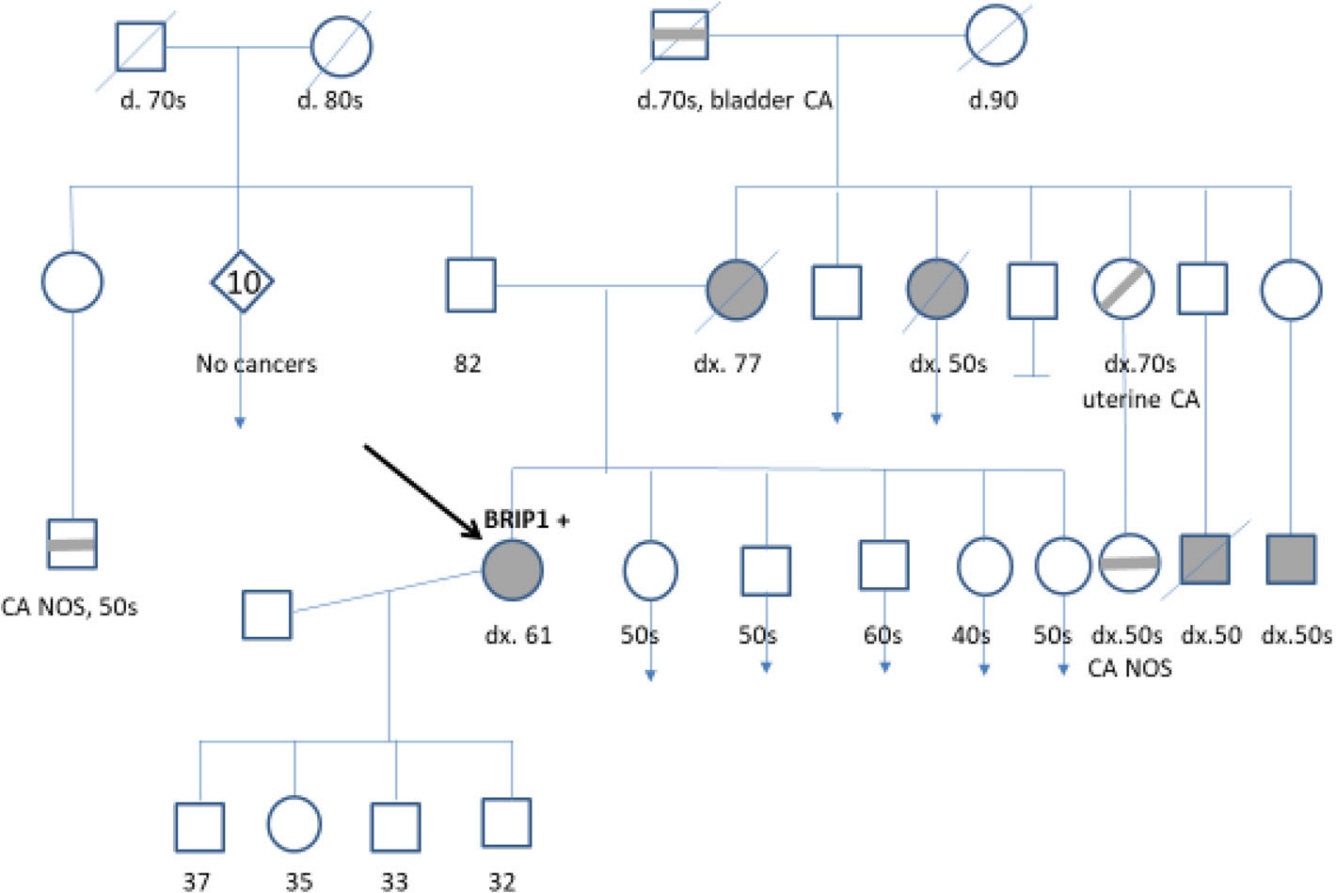
Family pedigree chart for case report 2 (see Fig. 2 for legend)

Sixty-two y/o female with a history of uterine fibroid, hysterectomy and salpingo-oophorectomy underwent screening colonoscopy which showed 4.9 × 3.4 cm circumferential mass in the proximal ascending colon. The patient did not have any gastrointestinal complaints of abdominal pain, constipation or blood in stools. Family history includes metastatic colon cancer in mother at age 77, maternal aunt diagnosed with colon cancer at age 50; another maternal aunt was diagnosed with uterine cancer in her 70’s. Two cousins on the maternal side had colon cancer at age 50. Pedigree chart for patient’s family history of cancer is shown below (Fig. 3). Physical examination was normal. Biopsy of colon mass showed moderately differentiated adenocarcinoma. Staging work up revealed two liver lesions. She underwent laparoscopic right colectomy and partial hepatectomy. Surgical specimen after hemicolectomy was sent for next-generation sequence analysis. Genomic alterations identified include *BRIP1 S988 fs: AKT1 E17K: mTOR E1799K: APC R1450: CREBBP S889: FAM123B R358*: *GNAS R201C: TP53 P177L* and *4213Q*. No reported alterations in *KRAS, NRAS*, and *BRAF*. Lynch syndrome screen by IHC (*MLH1, MSH2, MSH6* and *PMS2* proteins) was normal on the pathological tissue. The patient had a family history of colon cancer and wanted to be evaluated for gene mutations associated with hereditary cancer. Initial testing was done with commercially available 17 gene panel associated with colon cancer. Further germline testing for *BRIP1* gene mutation was done based on high allele frequency of *BRIP1* mutation in tumor tissue. This showed patient was heterozygous for *c.2962deIT* pathogenic mutation located in coding exon 19 of the *BRIP1* gene. She was staged as IVA given metastatic liver disease. She was treated with twelve cycles of chemotherapy FOLFOX. Two years later she was found to have a recurrence with liver lesions on PET CT. She underwent laparoscopic resection of hepatic metastases followed by adjuvant chemotherapy with eight cycles of FOLFIRI. Repeat scans did not show any active disease.

## Discussion and conclusions

Colorectal cancer (CRC) is the third most frequently diagnosed cancer and the second leading cause of cancer death in the United States [1]. Between 2 to 5% of all colon cancers arise in the setting of well-defined colon inherited syndromes whereas another 20% of colon cancers are associated with an increased familial incidence but not following classical transmission patterns. Hereditary Non-Polyposis Colorectal Cancer (HNPCC) or Lynch syndrome is caused by a mutation in one of the mismatch repair genes (*MSH2, MSH6, PMS2, MLH1, and EPCAM*) and is the most common form of genetically-determined colon cancer accounting for 2 to 4% of all CRC cases [4]. Familial adenomatosis polyposis (FAP) and *MUTYH* associated polyposis are other highly penetrant gene mutations causing recognizable syndromes. Other moderately penetrant gene mutations for colorectal cancer (with lifetime risks of cancer between 10 and 50%) include *BMPRA1, CHEK2, POLD1, POLE, PTEN, SMAD4, PTEN, CDH1,* and *STK11*. Patients found to have these pathogenic mutations along with personal or family history indicative of increased colon cancer risk, benefit from earlier or more frequent screening with colonoscopy and for other malignancies associated with that particular mutant gene. Information regarding any genetic mutation may result in a personalized plan for cancer prevention and early detection. Emerging evidence has identified additional genes that may be associated with an increased incidence of colon cancer [12].

Next generation sequence testing (NGS) and multi-gene “panel” germline mutation testing have provided tools to help identify additional genes associated with an increased risk of colon cancer [5]. NGS of tumor tissue can detect multiple types of genomic alterations, including nucleotide substitutions, small insertions and deletions, copy number variations and chromosomal rearrangements. NGS will identify mutations useful in treatment planning, e.g., analyses of *KRAS, NRAS, BRAF, HER2 neu* and microsatellite instability (MSI) status all have implications for treatment decisions. NGS will also identify additional mutations in tumor tissue whose clinical significance has not been clearly defined. However, some of the mutations seen in tumor tissue will be constitutional, and might not have been considered in the etiology of the patient’s tumor. As shown in these two patients, tumor genomics can help predict the presence of germline mutations [6]. A new paradigm in cancer genetics is thus to do paired genomic testing on tumor and peripheral blood and to consult the results of tumor genomics when deciding on which genes to test in the germline.

Both of our patients had risk factors which put them at higher likelihood of having an inherited cancer syndrome and were referred to a medical geneticist for assessment. The first patient had colon cancer diagnosed at the relatively young age of 52. The second patient had multiple family members with a history of colon cancer, albeit mainly at later ages. Both patients were initially tested for germline mutation with a commercially available multigene test which evaluates for genes commonly associated with hereditary colon cancer. These genes include *APC, BMPR1A, CDH1, CHEK2, MLH1, MSH2, MSH6, MUTYH, PMS2, POLD1, POLE, PTEN, SMAD4, STK11, TP53, EPCAM,* and *GREM1*. Both patients were negative for any of the above mutations in lymphocytes. Further testing was directed based on NGS test results. Both our patients had NGS testing of tumor tissue which was negative for *KRAS, NRAS, BRAF* mutations and demonstrated microsatellite stability. Our first patient had mutations in *BRIP1, TP53, APC,* and *CDK8* in tumor tissue. The second patient had mutations in *AKT1, MTOR, APC, BRIP1, CREBBP, FAM123B, GNAS,* and *TP53*. Both patients were tested for *BRIP1* germline mutation and were positive for the same pathogenic mutation identified in tumor tissue. The first patient had a deletion of one nucleotide at position 2962 located in exon 19 of the *BRIP1* gene, causing translational frameshift with a predicted alternate stop codon. The second patient had the insertion of a nucleotide at position 1853 located in coding exon 12 of the *BRIP1* gene causing translational frameshift with a predicted alternate stop codon. These alterations result in loss of function by premature protein truncation or mRNA decay. The importance of these germline mutations needs to be interpreted based on evidence of their association with the disease. These *BRIP1* mutations have enough evidence that they are pathogenic to cause an increased risk of ovarian and breast cancer [7]. Whether these are variants of unknown significance or pathogenic for colon cancer itself has yet to be established.

*BRCA-1* interacting (*BRIP1*) gene maps to chromosome 17 (17q23.2). It encodes a protein which is a member of RecQ DEAH helicase family and interacts with BRCT repeat of (*BRCA1*). The bound complex is essential in the normal double strand break repair function of the *BRCA1* complex. Monoallelic pathogenic germline mutations in *BRIP1* confer up to 10% cumulative risk of ovarian cancer [7]. Also, *BRIP1* mutations are associated with increased female breast cancer risk [8]. Biallelic pathogenic mutations in the *BRIP1* gene are known to cause Fanconi anemia type J (FA-J), a rare autosomal recessive disorder affecting multiple body systems. This syndrome is characterized by marrow failure and variable presentation of anomalies, including short stature, abnormal skin pigmentation, abnormal thumbs, malformations of the skeletal central nervous systems, and developmental delay. We propose a pathogenic role of BRIP-1 in causing colon cancer. *BRIP1* mutations have been reported in 3% of the colon cancer tissue samples analyzed in the colorectal adenocarcinoma TCGA (The cancer genome atlas) dataset [9]. There have also been studies where *BRIP1* has shown to be a factor in determining the recurrence rate in colon cancer. A 31 gene molecular signature which included *BRIP1* was developed using logistic regression analysis, and its presence was associated with increased recurrence of colon cancer [10]. It may be relevant that both patients here had recurrences. The increased *BRIP1* expression had been implicated in resistance to 5-fluorouracil treatment [11]. We hypothesize that germline *BRIP1* mutation was a factor in the etiology of the colon cancers in our patients.

There are current clinical implications of having tested positive for *BRIP1* germline pathogenic mutation. National comprehensive cancer network (NCCN) recommends consideration of prophylactic salpingo-oophorectomy to decrease the risk of developing ovarian cancer. Based on current evidence, this surgery should be considered around age 50–55 [11], The current NCCN guidelines do not recommend additional breast cancer screening for individuals with a single pathogenic *BRIP1* variant but caution that management should ultimately be guided by personal and family history. In our opinion patients who have *BRIP1* mutation may potentially benefit from earlier or more frequent screening with colonoscopy, especially with family history of colon cancer. NCCN recognized the role of newly identified genes that may be associated with an increased risk of colon cancer [12]. Our case reports suggest that *BRIP1* is another similar gene which may have a causative role in colon cancer. Larger studies will provide more evidence to ascertain if this association is strong. This information can be obtained with the addition of the *BRIP1* gene to research level panels evaluating germline mutations and colon cancer predisposition. In conclusion, our case reports suggest that germline *BRIP1* mutation may be associated with colon cancer predisposition and should be further investigated.

## Abbreviations

BRCA1: Breast Cancer gene 1
BRIP1: BRCA1 Interacting Protein C- terminal helicase 1
CRC: Colorectal cancer
CT scan: Computer tomography scan
FOLFIRI: 5-fluorouracil, Leucovorin, Irinotecan
FOLFOX: 5-fluorouracil, Leucovorin, oxaliplatin
HIPEC: Hyperthermic intraperitoneal chemotherapy.
MSI: Microsatellite instability
NCCN: National comprehensive cancer network
NGS: Next generation sequence
PET CT: Positron emission tomography
TCGA: The cancer genome atlas dataset
